# Supplementary figures and images for: Distinct responses to rare codons in select Drosophila tissues
Source: eLife. 2022 May 6;11:e76893. doi: 10.7554/eLife.76893 (PMC9116940; doi:10.7554/eLife.76893)

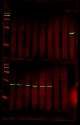

Supplement: Figure 1—figure supplement 1—source data 1. — Blot image was taken in the 800 nm channel and indicates the signal from anti-GFP antibody recognizing transgenic reporter derived GFP protein. Ladder is LI-COR Chameleon Duo pre-stained protein ladder. [file elife-76893-fig1-figsupp1-data1.zip › 0000181_01/0000181_01_TH.jpg]

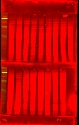

Supplement: Figure 1—figure supplement 1—source data 2. — Blot image was taken in the 700 nm channel and indicates the total protein stain obtained using the LI-COR Revert700 Total Protein Stain kit. Ladder is LI-COR Chameleon Duo pre-stained protein ladder. [file elife-76893-fig1-figsupp1-data2.zip › 0000179_01/0000179_01_TH.jpg]

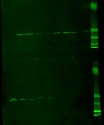

Supplement: Figure 1—figure supplement 1—source data 3. — Blot image was taken in the 800 nm channel and indicates the signal from anti-GFP antibody recognizing transgenic reporter derived GFP protein. Ladder is LI-COR Chameleon Duo pre-stained protein ladder. [file elife-76893-fig1-figsupp1-data3.zip › 0000187_01/0000187_01_TH.jpg]

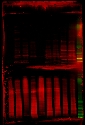

Supplement: Figure 1—figure supplement 1—source data 4. — Blot image was taken in the 700 nm channel and indicates the total protein stain obtained using the LI-COR Revert700 Total Protein Stain kit. Ladder is LI-COR Chameleon Duo pre-stained protein ladder. [file elife-76893-fig1-figsupp1-data4.zip › 0000183_01/0000183_01_TH.jpg]

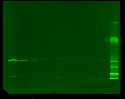

Supplement: Figure 1—figure supplement 1—source data 5. — Blot image was taken in the 800 nm channel and indicates the signal from anti-GFP antibody recognizing transgenic reporter derived GFP protein. Ladder is LI-COR Chameleon Duo pre-stained protein ladder. [file elife-76893-fig1-figsupp1-data5.zip › 0000200_01/0000200_01_TH.jpg]

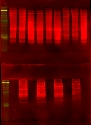

Supplement: Figure 1—figure supplement 1—source data 6. — Blot image was taken in the 700 nm channel and indicates the total protein stain obtained using the LI-COR Revert700 Total Protein Stain kit. Ladder is LI-COR Chameleon Duo pre-stained protein ladder. [file elife-76893-fig1-figsupp1-data6.zip › 0000193_01/0000193_01_TH.jpg]

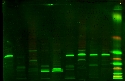

Supplement: Figure 3—figure supplement 1—source data 1. — Blot image was taken in the 800 nm channel and indicates the signal from anti-GFP antibody recognizing transgenic reporter derived GFP protein. Ladder is LI-COR Chameleon Duo pre-stained protein ladder. [file elife-76893-fig3-figsupp1-data1.zip › 0000208_01/0000208_01_TH.jpg]

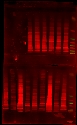

Supplement: Figure 3—figure supplement 1—source data 2. — Blot image was taken in the 700 nm channel and indicates the total protein stain obtained using the LI-COR Revert700 Total Protein Stain kit. Ladder is LI-COR Chameleon Duo pre-stained protein ladder. [file elife-76893-fig3-figsupp1-data2.zip › 0000204_01/0000204_01_TH.jpg]

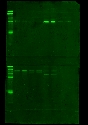

Supplement: Figure 3—figure supplement 1—source data 3. — Blot image was taken in the 800 nm channel and indicates the signal from anti-GFP antibody recognizing transgenic reporter derived GFP protein. Ladder is LI-COR Chameleon Duo pre-stained protein ladder. [file elife-76893-fig3-figsupp1-data3.zip › 0000202_01/0000202_01_TH.jpg]

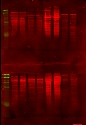

Supplement: Figure 3—figure supplement 1—source data 4. — Blot image was taken in the 700 nm channel and indicates the total protein stain obtained using the LI-COR Revert700 Total Protein Stain kit. Ladder is LI-COR Chameleon Duo pre-stained protein ladder. [file elife-76893-fig3-figsupp1-data4.zip › 0000198_01/0000198_01_TH.jpg]

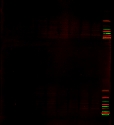

Supplement: Figure 5—figure supplement 1—source data 1. — Blot image was taken in the 800 nm channel and indicates the signal from anti-FLAG M2 antibody recognizing FLAG-tagged transgenic RpL10Aa reporter derived protein. Ladder is LI-COR Chameleon Duo pre-stained protein ladder. [file elife-76893-fig5-figsupp1-data1.zip › 0000216_01/0000216_01_TH.jpg]

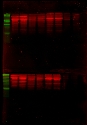

Supplement: Figure 5—figure supplement 1—source data 2. — Blot image was taken in the 700 nm channel and indicates the total protein stain obtained using the LI-COR Revert700 Total Protein Stain kit. Ladder is LI-COR Chameleon Duo pre-stained protein ladder. [file elife-76893-fig5-figsupp1-data2.zip › 0000212_01/0000212_01_TH.jpg]

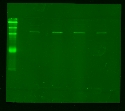

Supplement: Figure 5—figure supplement 2—source data 1. — Blot image was taken in the 800 nm channel and indicates the signal from anti-FLAG M2 antibody recognizing FLAG-tagged transgenic RpL10Aa reporter derived protein. Ladder is LI-COR Chameleon Duo pre-stained protein ladder. [file elife-76893-fig5-figsupp2-data1.zip › 0000201_01/0000201_01_TH.jpg]

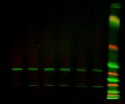

Supplement: Figure 5—figure supplement 2—source data 3. — Blot image was taken in the 800 nm channel and indicates the signal from anti-FLAG M2 antibody recognizing FLAG-tagged transgenic RpL10Aa reporter derived protein. Ladder is LI-COR Chameleon Duo pre-stained protein ladder. [file elife-76893-fig5-figsupp2-data3.zip › 0000206_01/0000206_01_TH.jpg]
