# Supplementary material for: Distinct responses to rare codons in select Drosophila tissues
Source: eLife. 2022 May 6;11:e76893. doi: 10.7554/eLife.76893 (PMC9116940; doi:10.7554/eLife.76893)
Supplement: Supplementary file 1. [file elife-76893-supp1.docx]

| **Reporter**  **Supplementary File 1 – Full coding sequences of reporters.** | **Full CDS** |
| --- | --- |
| **GFP0D** | ATGAGCAAGGGCGAGGAGCTGTTCACCGGCGTGGTGCCCATCCTGGTGGAGCTGGATGGCGATGTGAACGGCCACAAGTTCAGCGTGAGCGGCGAGGGCGAGGGCGATGCCACCTACGGCAAGCTGACCCTGAAGTTCATCTGCACCACCGGCAAGCTGCCCGTGCCCTGGCCCACCCTGGTGACCACCCTGACCTACGGCGTGCAGTGCTTCAGCCGCTACCCCGATCACATGAAGCAGCACGATTTCTTCAAGAGCGCCATGCCCGAGGGCTACGTGCAGGAGCGCACCATCTTCTTCAAGGATGATGGCAACTACAAGACCCGCGCCGAGGTGAAGTTCGAGGGCGATACCCTGGTGAACCGCATCGAGCTGAAGGGCATCGATTTCAAGGAGGATGGCAACATCCTGGGCCACAAGCTGGAGTACAACTACAACAGCCACAACGTGTACATCATGGCCGATAAGCAGAAGAACGGCATCAAGGTGAACTTCAAGATCCGCCACAACATCGAGGATGGCAGCGTGCAGCTGGCCGATCACTACCAGCAGAACACCCCCATCGGCGATGGCCCCGTGCTGCTGCCCGATAACCACTACCTGAGCACCCAGAGCGCCCTGAGCAAGGATCCCAACGAGAAGCGCGATCACATGGTGCTGCTGGAGTTCGTGACCGCCGCCGGCATCACCCTGGGCATGGATGAGCTGTACAAGTAA |
| **GFP30D** | ATGTCTAAAGGTGAGGAGCTCTTCACTGGTGTAGTTCCCATACTAGTGGAACTGGACGGAGACGTCAACGGCCATAAATTCTCAGTGTCGGGCGAAGGTGAAGGAGATGCCACATACGGAAAACTGACGCTTAAGTTTATTTGTACAACTGGGAAGCTGCCAGTCCCCTGGCCAACACTGGTTACTACTCTAACCTACGGCGTGCAATGTTTTAGTCGCTATCCGGACCACATGAAACAACACGATTTCTTTAAAAGCGCGATGCCTGAGGGGTACGTGCAGGAAAGGACGATATTTTTTAAGGATGATGGAAACTATAAAACGCGAGCCGAGGTCAAGTTTGAGGGCGACACCCTTGTGAATCGGATTGAACTGAAGGGTATTGACTTTAAGGAAGATGGAAATATCCTGGGACACAAGTTGGAGTATAATTACAACTCCCATAACGTGTATATCATGGCTGACAAACAGAAGAATGGCATCAAAGTGAACTTTAAGATCCGTCATAACATCGAGGACGGCTCCGTGCAGTTGGCGGATCATTACCAGCAAAATACCCCAATAGGTGATGGCCCAGTTTTGCTGCCGGATAACCATTACCTAAGCACCCAATCCGCACTTTCGAAGGATCCGAATGAAAAGCGCGACCACATGGTATTGCTCGAATTCGTCACCGCTGCTGGCATCACGTTAGGCATGGATGAGTTATATAAGTAG |
| **GFP50D** | ATGTCTAAAGGTGAGGAACTCTTCACTGGGGTCGTTCCTATACTAGTGGAATTAGACGGAGACGTCAACGGACATAAATTTTCAGTGTCGGGCGAAGGTGAAGGAGATGCGACATATGGAAAATTAACACTTAAATTTATTTGTACAACTGGGAAGCTCCCTGTCCCTTGGCCGACTTTAGTTACTACTCTAACCTACGGCGTCCAATGTTTTAGTAGATATCCGGACCACATGAAACAGCATGATTTTTTTAAATCCGCAATGCCTGAAGGGTATGTGCAGGAAAGGACGATATTTTTTAAAGATGATGGAAACTATAAAACGCGAGCCGAGGTCAAATTTGAAGGCGACACGTTAGTGAATCGGATAGAATTAAAAGGTATTGACTTTAAGGAAGATGGAAATATTTTAGGACACAAATTGGAGTATAATTATAACAGCCATAACGTGTATATCATGGCAGACAAGCAGAAAAATGGGATAAAAGTGAACTTTAAAATCCGCCATAACATCGAAGACGGCTCCGTGCAATTGGCAGATCATTACCAACAAAATACTCCAATAGGAGATGGTCCAGTTCTTCTGCCGGATAACCATTATTTAAGCACCCAATCCGCATTATCGAAGGACCCGAATGAAAAGCGTGACCATATGGTATTACTGGAATTTGTAACCGCTGCAGGAATAACCTTGGGAATGGACGAATTGTATAAGTGA |
| **GFP60Dv1** | ATGTCTAAAGGGGAGGAGTTATTTACGGGGGTAGTACCCATTTTAGTAGAACTCGACGGTGATGTAAATGGGCATAAATTCTCTGTATCTGGAGAGGGGGAGGGGGACGCCACATACGGGAAATTAACTTTGAAATTCATATGTACCACTGGCAAGTTACCTGTACCTTGGCCGACGTTAGTAACCACTCTTACGTATGGGGTTCAATGTTTTTCCAGATATCCTGACCACATGAAACAACACGACTTTTTCAAATCCGCCATGCCTGAAGGATATGTACAGGAGAGAACGATATTTTTTAAAGACGACGGGAATTATAAGACAAGGGCAGAAGTGAAATTCGAGGGGGACACTCTAGTAAACAGAATTGAGTTAAAAGGAATAGATTTCAAGGAAGACGGGAATATACTGGGGCACAAGCTTGAGTACAACTATAATTCCCACAACGTCTACATTATGGCAGACAAGCAGAAAAACGGGATAAAAGTCAATTTTAAAATCCGGCACAATATAGAAGACGGGTCTGTACAACTTGCCGATCATTATCAACAGAATACTCCTATCGGGGACGGGCCGGTACTCCTACCTGACAATCACTATTTATCTACTCAATCGGCATTATCTAAAGACCCTAATGAAAAAAGAGACCACATGGTATTATTAGAGTTTGTGACTGCAGCTGGTATTACCTTAGGGATGGACGAACTATATAAATAA |
| **GFP60Dv2** | ATGTCGAAAGGTGAGGAGCTCTTTACTGGAGTAGTACCAATATTAGTAGAATTAGACGGGGATGTCAATGGTCACAAATTTTCGGTAAGTGGGGAGGGTGAAGGGGACGCAACGTACGGGAAATTAACTTTAAAATTTATATGCACGACTGGAAAATTGCCTGTACCTTGGCCTACTTTAGTAACCACTTTAACGTACGGGGTACAATGCTTCAGTAGATATCCAGATCACATGAAACAACACGATTTCTTTAAATCTGCAATGCCTGAGGGGTACGTACAAGAACGAACTATATTCTTTAAAGACGACGGGAATTACAAGACTCGTGCGGAAGTAAAATTCGAAGGGGATACTCTGGTAAACAGAATTGAATTAAAAGGCATAGATTTCAAAGAAGATGGGAACATTCTCGGTCATAAACTCGAATATAATTATAATTCTCACAATGTATACATAATGGCGGACAAACAGAAAAATGGTATAAAAGTTAACTTTAAGATTAGACATAATATAGAAGACGGGTCTGTACAGTTAGCGGATCACTATCAACAAAATACTCCAATAGGGGACGGTCCAGTACTCCTCCCAGACAACCATTACTTATCTACTCAAAGTGCGTTATCTAAAGACCCTAATGAGAAACGGGATCATATGGTACTCCTCGAGTTTGTAACTGCAGCGGGGATAACTTTAGGTATGGATGAACTGTACAAATGA |
| **GFP60Dv3** | ATGTCGAAAGGGGAAGAGTTATTCACGGGAGTAGTACCTATATTAGTAGAATTGGACGGAGACGTCAACGGGCATAAATTTTCTGTTAGTGGAGAAGGTGAGGGGGACGCAACTTATGGTAAGTTAACGTTAAAATTCATTTGTACTACTGGGAAATTACCTGTCCCATGGCCTACTTTAGTAACTACTTTAACGTATGGGGTCCAATGTTTTAGCAGATACCCAGACCATATGAAACAGCACGACTTTTTTAAAAGTGCTATGCCTGAGGGGTACGTTCAGGAACGAACGATTTTCTTTAAAGATGATGGGAATTACAAAACTAGAGCAGAGGTTAAGTTTGAAGGGGACACTTTAGTAAACAGAATTGAACTCAAAGGTATAGATTTCAAAGAAGATGGGAATATTCTGGGTCACAAATTAGAGTACAATTATAATAGTCATAACGTATACATTATGGCCGACAAACAAAAAAACGGTATTAAAGTAAATTTCAAGATAAGACATAATATTGAAGACGGGAGTGTACAATTAGCAGATCACTATCAACAAAATACTCCAATAGGGGACGGGCCTGTACTCCTCCCTGACAATCATTATTTATCTACTCAGTCTGCGCTCTCTAAAGACCCTAACGAAAAACGCGATCACATGGTTTTATTAGAGTTCGTAACTGCAGCAGGGATAACTCTCGGGATGGATGAGTTATACAAGTGA |
| **GFP70D** | ATGTCTAAAGGGGAGGAATTATTTACCGGGGTAGTACCTATATTAGTGGAACTTGACGGGGATGTAAATGGGCATAAATTCTCTGTATCTGGGGAAGGGGAAGGTGATGCCACATATGGGAAACTTACGCTTAAATTTATCTGTACCACTGGGAAATTACCTGTACCTTGGCCCACCTTAGTAACTACTTTAACTTACGGTGTACAGTGCTTTAGCAGATATCCAGACCATATGAAGCAGCATGACTTCTTTAAATCTGCAATGCCAGAAGGCTATGTTCAGGAAAGAACTATATTTTTTAAAGATGACGGGAATTATAAGACTAGAGCAGAAGTTAAATTCGAAGGCGACACTTTAGTCAATCGGATAGAATTAAAAGGGATCGATTTTAAAGAAGATGGCAACATACTCGGGCATAAGTTAGAATATAATTATAACTCGCACAATGTATATATCATGGCAGACAAACAAAAAAATGGGATCAAAGTAAATTTTAAGATACGACATAATATAGAAGACGGGTCTGTACAACTAGCAGATCATTACCAACAGAATACTCCCATTGGTGACGGACCTGTATTATTACCTGATAACCATTACTTATCTACTCAGTCTGCATTAAGTAAAGACCCTAATGAAAAAAGAGACCATATGGTATTATTAGAATTTGTAACTGCAGCAGGGATCACTTTAGGGATGGACGAGTTATATAAATGA |
| **GFP80D** | ATGTCCAAAGGCGAGGAATTATTTACGGGGGTAGTACCTATATTAGTAGAGTTAGACGGAGACGTGAATGGGCATAAGTTTTCTGTATCTGGGGAAGGGGAAGGGGACGCAACTTATGGGAAATTAACTTTAAAGTTTATATGTACTACTGGGAAATTACCTGTACCCTGGCCTACATTAGTAACTACCTTAACATATGGGGTACAATGCTTTTCTAGATATCCTGACCATATGAAACAGCATGACTTTTTTAAGTCTGCAATGCCTGAGGGGTATGTACAGGAAAGAACAATATTTTTTAAAGACGACGGGAATTACAAAACTAGAGCAGAAGTAAAATTTGAAGGGGATACTTTAGTAAATCGTATAGAACTTAAAGGGATAGACTTTAAAGAAGACGGGAATATATTAGGGCATAAGTTAGAATATAATTATAATAGCCATAATGTTTACATAATGGCAGACAAGCAAAAAAATGGGATAAAAGTAAATTTTAAAATAAGACATAATATAGAAGACGGGTCTGTACAATTAGCAGACCATTATCAGCAAAATACTCCTATAGGGGACGGGCCTGTATTATTACCCGATAACCATTATCTCTCCACTCAATCGGCATTGTCAAAAGACCCTAATGAAAAAAGAGACCATATGGTACTCTTAGAGTTTGTAACTGCAGCAGGGATCACTTTAGGGATGGATGAATTATATAAATGA |
| **GFP90D** | ATGTCTAAAGGGGAGGAATTATTTACTGGGGTAGTACCTATTTTAGTAGAATTAGACGGGGACGTAAATGGGCATAAATTTTCTGTATCTGGGGAAGGGGAAGGGGACGCAACTTATGGGAAATTAACTTTAAAATTTATATGTACTACTGGGAAATTACCTGTACCTTGGCCGACTTTAGTAACTACTTTAACTTATGGGGTACAATGTTTTAGCAGATATCCTGACCACATGAAACAACACGACTTTTTTAAATCTGCAATGCCTGAAGGGTATGTACAGGAGAGAACTATATTTTTTAAAGACGACGGGAATTATAAAACTAGAGCAGAAGTAAAATTTGAGGGGGACACTTTAGTAAATAGAATAGAATTAAAAGGCATAGACTTCAAAGAAGACGGGAATATATTAGGGCATAAATTGGAATATAATTATAATTCTCATAATGTATACATAATGGCAGACAAACAAAAAAATGGGATAAAAGTAAATTTTAAAATAAGACATAATATAGAAGACGGGTCTGTACAACTTGCAGACCATTATCAACAAAATACTCCTATAGGGGACGGGCCTGTATTATTACCTGACAATCATTATTTATCTACTCAAAGTGCATTATCTAAAGACCCTAATGAAAAAAGAGACCACATGGTATTATTAGAATTTGTAACTGCAGCAGGGATAACTTTAGGGATGGACGAACTTTATAAATGA |
| **GFP100D** | ATGTCTAAAGGGGAAGAATTATTTACTGGGGTAGTACCTATATTAGTAGAATTAGACGGGGACGTAAATGGGCATAAATTTTCTGTATCTGGGGAAGGGGAAGGGGACGCAACTTATGGGAAATTAACTTTAAAATTTATATGTACTACTGGGAAATTACCTGTACCTTGGCCTACTTTAGTAACTACTTTAACTTATGGGGTACAATGTTTTTCTAGATATCCTGACCATATGAAACAACATGACTTTTTTAAATCTGCAATGCCTGAAGGGTATGTACAAGAAAGAACTATATTTTTTAAAGACGACGGGAATTATAAAACTAGAGCAGAAGTAAAATTTGAAGGGGACACTTTAGTAAATAGAATAGAATTAAAAGGGATAGACTTTAAAGAAGACGGGAATATATTAGGGCATAAATTAGAATATAATTATAATTCTCATAATGTATATATAATGGCAGACAAACAAAAAAATGGGATAAAAGTAAATTTTAAAATAAGACATAATATAGAAGACGGGTCTGTACAATTAGCAGACCATTATCAACAAAATACTCCTATAGGGGACGGGCCTGTATTATTACCTGACAATCATTATTTATCTACTCAATCTGCATTATCTAAAGACCCTAATGAAAAAAGAGACCATATGGTATTATTAGAATTTGTAACTGCAGCAGGGATAACTTTAGGGATGGACGAATTATATAAATAA |
| **GFP50C3’** | ATGTCGAAGGGAGAGGAGTTGTTCACAGGTGTCGTCCCGATCCTAGTCGAGCTAGATGGTGATGTTAACGGACACAAGTTCTCGGTCTCCGGTGAGGGAGAGGGCGATGCTACGTACGGAAAGCTAACACTAAAGTTCATTTGCACGACAGGTAAGCTCCCGGTGCCGTGGCCAACGTTGGTCACGACGCTCACATACGGCGTTCAGTGCTTCTCCCGGTACCCGGATCACATGAAGCAGCACGATTTCTTCAAGTCCGCCATGCCAGAGGGATACGTCCAGGAGCGTACGATTTTCTTCAAGGATGATGGAAACTACAAGACGCGGGCTGAGGTCAAGTTCGAGGGTGATACGCTCGTTAATAGAATAGAATTAAAAGGGATAGACTTTAAAGAAGACGGGAATATATTAGGGCATAAATTAGAATATAATTATAATTCTCATAATGTATATATAATGGCAGACAAACAAAAAAATGGGATAAAAGTAAATTTTAAAATAAGACATAATATAGAAGACGGGTCTGTACAATTAGCAGACCATTATCAACAAAATACTCCTATAGGGGACGGGCCTGTATTATTACCTGACAATCATTATTTATCTACTCAATCTGCATTATCTAAAGACCCTAATGAAAAAAGAGACCATATGGTATTATTAGAATTTGTAACTGCAGCAGGGATAACTTTAGGGATGGACGAATTATATAAATGA |
| **GFP60C3’** | ATGTCGAAGGGTGAGGAGTTGTTCACGGGTGTGGTTCCGATTCTCGTTGAGCTCGATGGTGATGTTAACGGCCACAAGTTCAGCGTTTCGGGAGAGGGTGAGGGCGATGCTACGTACGGAAAGCTGACCTTGAAGTTCATTTGCACGACGGGAAAGCTGCCGGTCCCGTGGCCAACGCTGGTCACGACGTTGACGTACGGAGTGCAGTGCTTCTCGCGTTACCCGGATCACATGAAGCAGCACGATTTCTTCAAGTCGGCGATGCCGGAGGGTTACGTGCAAGAAAGAACTATATTTTTTAAAGACGACGGGAATTATAAAACTAGAGCAGAAGTAAAATTTGAAGGGGACACTTTAGTAAATAGAATAGAATTAAAAGGGATAGACTTTAAAGAAGACGGGAATATATTAGGGCATAAATTAGAATATAATTATAATTCTCATAATGTATATATAATGGCAGACAAACAAAAAAATGGGATAAAAGTAAATTTTAAAATAAGACATAATATAGAAGACGGGTCTGTACAATTAGCAGACCATTATCAACAAAATACTCCTATAGGGGACGGGCCTGTATTATTACCTGACAATCATTATTTATCTACTCAATCTGCATTATCTAAAGACCCTAATGAAAAAAGAGACCATATGGTATTATTAGAATTTGTAACTGCAGCAGGGATAACTTTAGGGATGGACGAATTATATAAATGA |
| **GFP70C3’** | ATGAGCAAGGGCGAGGAGCTGTTCACGGGTGTGGTCCCCATTCTGGTGGAGCTGGATGGCGATGTTAACGGACACAAGTTCAGTGTTAGCGGAGAGGGTGAGGGCGATGCCACGTACGGCAAGCTGACCCTAAAGTTCATCTGCACCACCGGCAAGCTCCCAGTGCCGTGGCCGACGCTAGTTACCACCCTGACCTACGGTGTTCAATGTTTTTCTAGATATCCTGACCATATGAAACAACATGACTTTTTTAAATCTGCAATGCCTGAAGGGTATGTACAAGAAAGAACTATATTTTTTAAAGACGACGGGAATTATAAAACTAGAGCAGAAGTAAAATTTGAAGGGGACACTTTAGTAAATAGAATAGAATTAAAAGGGATAGACTTTAAAGAAGACGGGAATATATTAGGGCATAAATTAGAATATAATTATAATTCTCATAATGTATATATAATGGCAGACAAACAAAAAAATGGGATAAAAGTAAATTTTAAAATAAGACATAATATAGAAGACGGGTCTGTACAATTAGCAGACCATTATCAACAAAATACTCCTATAGGGGACGGGCCTGTATTATTACCTGACAATCATTATTTATCTACTCAATCTGCATTATCTAAAGACCCTAATGAAAAAAGAGACCATATGGTATTATTAGAATTTGTAACTGCAGCAGGGATAACTTTAGGGATGGACGAATTATATAAATGA |
| **GFP80C3’** | ATGTCCAAGGGCGAGGAGTTGTTCACGGGTGTGGTTCCAATTCTCGTCGAGCTGGATGGTGATGTTAACGGTCACAAGTTCAGCGTGTCAGGCGAGGGCGAGGGCGATGCCACGTACGGCAAGTTGACTTTAAAATTTATATGTACTACTGGGAAATTACCTGTACCTTGGCCTACTTTAGTAACTACTTTAACTTATGGGGTACAATGTTTTTCTAGATATCCTGACCATATGAAACAACATGACTTTTTTAAATCTGCAATGCCTGAAGGGTATGTACAAGAAAGAACTATATTTTTTAAAGACGACGGGAATTATAAAACTAGAGCAGAAGTAAAATTTGAAGGGGACACTTTAGTAAATAGAATAGAATTAAAAGGGATAGACTTTAAAGAAGACGGGAATATATTAGGGCATAAATTAGAATATAATTATAATTCTCATAATGTATATATAATGGCAGACAAACAAAAAAATGGGATAAAAGTAAATTTTAAAATAAGACATAATATAGAAGACGGGTCTGTACAATTAGCAGACCATTATCAACAAAATACTCCTATAGGGGACGGGCCTGTATTATTACCTGACAATCATTATTTATCTACTCAATCTGCATTATCTAAAGACCCTAATGAAAAAAGAGACCATATGGTATTATTAGAATTTGTAACTGCAGCAGGGATAACTTTAGGGATGGACGAATTATATAAATGA |
| **GFP90C3’** | ATGAGCAAGGGCGAGGAGCTATTCACGGGTGTGGTGCCAATTTTGGTGGAGCTGGACGGGGACGTAAATGGGCATAAATTTTCTGTATCTGGGGAAGGGGAAGGGGACGCAACTTATGGGAAATTAACTTTAAAATTTATATGTACTACTGGGAAATTACCTGTACCTTGGCCTACTTTAGTAACTACTTTAACTTATGGGGTACAATGTTTTTCTAGATATCCTGACCATATGAAACAACATGACTTTTTTAAATCTGCAATGCCTGAAGGGTATGTACAAGAAAGAACTATATTTTTTAAAGACGACGGGAATTATAAAACTAGAGCAGAAGTAAAATTTGAAGGGGACACTTTAGTAAATAGAATAGAATTAAAAGGGATAGACTTTAAAGAAGACGGGAATATATTAGGGCATAAATTAGAATATAATTATAATTCTCATAATGTATATATAATGGCAGACAAACAAAAAAATGGGATAAAAGTAAATTTTAAAATAAGACATAATATAGAAGACGGGTCTGTACAATTAGCAGACCATTATCAACAAAATACTCCTATAGGGGACGGGCCTGTATTATTACCTGACAATCATTATTTATCTACTCAATCTGCATTATCTAAAGACCCTAATGAAAAAAGAGACCATATGGTATTATTAGAATTTGTAACTGCAGCAGGGATAACTTTAGGGATGGACGAATTATATAAATGA |
| **GFP50C5’** | ATGTCTAAAGGGGAAGAATTATTTACTGGGGTAGTACCTATATTAGTAGAATTAGACGGGGACGTAAATGGGCATAAATTTTCTGTATCTGGGGAAGGGGAAGGGGACGCAACTTATGGGAAATTAACTTTAAAATTTATATGTACTACTGGGAAATTACCTGTACCTTGGCCTACTTTAGTAACTACTTTAACTTATGGGGTACAATGTTTTTCTAGATATCCTGACCATATGAAACAACATGACTTTTTTAAATCTGCAATGCCTGAAGGGTATGTACAAGAAAGAACTATATTTTTTAAAGACGACGGGAATTATAAAACTAGAGCAGAAGTAAAATTTGAAGGGGACACTTTAGTAAACCGTATTGAGTTGAAGGGAATTGATTTCAAGGAGGATGGTAACATTCTTGGACACAAGTTGGAGTACAACTACAACTCCCACAACGTTTACATTATGGCTGATAAGCAGAAGAACGGTATTAAGGTCAACTTCAAGATTAGGCACAACATTGAGGATGGATCGGTCCAGCTCGCGGATCACTACCAGCAGAACACGCCAATTGGTGATGGTCCAGTTCTTCTTCCAGATAACCACTACTTGTCGACGCAGTCCGCTCTTTCGAAGGATCCGAACGAGAAGCGTGATCACATGGTTCTCTTGGAGTTCGTTACAGCTGCGGGAATTACGCTTGGTATGGATGAGCTCTACAAGTAG |
| **GFP54C3’** | ATGAGCAAGGGCGAGGAGCTGTTCACCGGCGTGGTGCCCATCCTGGTGGAGCTGGATGGCGATGTGAACGGCCACAAGTTCAGCGTGAGCGGCGAGGGCGAGGGCGATGCCACCTACGGCAAGCTGACCCTGAAGTTCATCTGCACCACCGGCAAGCTGCCCGTGCCCTGGCCCACCCTGGTGACCACCCTGACCTACGGCGTGCAGTGCTTCAGCCGCTACCCCGATCACATGAAGCAGCACGATTTCTTCAAGAGCGCCATGCCCGAGGGCTACGTGCAGGAGCGCACCATCTTCTTCAAGGATGATGGCAACTACAAGACCCGCGCCGAAGTAAAATTTGAAGGGGACACTTTAGTAAATAGAATAGAATTAAAAGGGATAGACTTTAAAGAAGACGGGAATATATTAGGGCATAAATTAGAATATAATTATAATTCTCATAATGTATATATAATGGCAGACAAACAAAAAAATGGGATAAAAGTAAATTTTAAAATAAGACATAATATAGAAGACGGGTCTGTACAATTAGCAGACCATTATCAACAAAATACTCCTATAGGGGACGGGCCTGTATTATTACCTGACAATCATTATTTATCTACTCAATCTGCATTATCTAAAGACCCTAATGAAAAAAGAGACCATATGGTATTATTAGAATTTGTAACTGCAGCAGGGATAACTTTAGGGATGGACGAATTATATAAATGA |
| **mGFP100Dv1** | ATGGTGAGCAAGGGCGAGGAGGATAACATGGCCATCATCAAGGAGTTCATGCGCTTCAAGGTGCACATGGAGGGCTCCGTGAACGGCCACGAGTTCGAGATCGAGGGCGAGGGCGAGGGCCGCCCCTACGAGGGCACCCAGACCGCCAAGCTGAAGGTGACCAAGGGTGGCCCCCTGCCCTTCGCCTGGGACATCCTGTCCCCTCAGTTCATGTACGGCTCCAAGGCCTACGTGAAGCACCCCGCCGACATCCCCGACTACTTGAAGCTGTCCTTCCCCGAGGGCTTCAAGTGGGAGCGCGTGATGAACTTCGAGGACGGCGGCGTGGTGACCGTGACCCAGGACTCCTCCCTGCAGGACGGCGAGTTCATCTACAAGGTGAAGCTGCGCGGCACCAACTTCCCCTCCGACGGCCCCGTAATGCAGAAGAAGACCATGGGCTGGGAGGCCTCCTCCGAGCGGATGTACCCCGAGGACGGCGCCCTGAAGGGCGAGATCAAGCAGAGGCTGAAGCTGAAGGACGGCGGCCACTACGACGCTGAGGTCAAGACCACCTACAAGGCCAAGAAGCCCGTGCAGCTGCCCGGCGCCTACAACGTCAACATCAAGTTGGACATCACCTCCCACAACGAGGACTACACCATCGTGGAACAGTACGAACGCGCCGAGGGCCGCCACTCCACCGGCGGCATGGACGAGCTGTACAAGGGCGGCGGCGGCAGCGGCGGCGGCGGCAGCGGCGGCGGCGGCAGCGGCGGCGGCGGCAGCATGTCTAAAGGGGAAGAATTATTTACTGGGGTAGTACCTATATTAGTAGAATTAGACGGGGACGTAAATGGGCATAAATTTTCTGTATCTGGGGAAGGGGAAGGGGACGCAACTTATGGGAAATTAACTTTAAAATTTATATGTACTACTGGGAAATTACCTGTACCTTGGCCTACTTTAGTAACTACTTTAACTTATGGGGTACAATGTTTTTCTAGATATCCTGACCATATGAAACAACATGACTTTTTTAAATCTGCAATGCCTGAAGGGTATGTACAAGAAAGAACTATATTTTTTAAAGACGACGGGAATTATAAAACTAGAGCAGAAGTAAAATTTGAAGGGGACACTTTAGTAAATAGAATAGAATTAAAAGGGATAGACTTTAAAGAAGACGGGAATATATTAGGGCATAAATTAGAATATAATTATAATTCTCATAATGTATATATAATGGCAGACAAACAAAAAAATGGGATAAAAGTAAATTTTAAAATAAGACATAATATAGAAGACGGGTCTGTACAATTAGCAGACCATTATCAACAAAATACTCCTATAGGGGACGGGCCTGTATTATTACCTGACAATCATTATTTATCTACTCAATCTGCATTATCTAAAGACCCTAATGAAAAAAGAGACCATATGGTATTATTAGAATTTGTAACTGCAGCAGGGATAACTTTAGGGATGGACGAATTATATAAATAA |
| **mGFP100Dv2** | ATGGTCTCCAAAGGAGAAGAAGACAATATGGCTATTATTAAAGAATTTATGCGTTTTAAAGTCCATATGGAAGGAAGCGTCAATGGACATGAATTTGAAATTGAAGGAGAAGGAGAAGGCCGCCCCTACGAGGGCACCCAGACCGCCAAGCTGAAGGTGACCAAGGGTGGCCCCCTGCCCTTCGCCTGGGACATCCTGTCCCCTCAGTTCATGTACGGCTCCAAGGCCTACGTGAAGCACCCCGCCGACATCCCCGACTACTTGAAGCTGTCCTTCCCCGAGGGCTTCAAGTGGGAGCGCGTGATGAACTTCGAGGACGGCGGCGTGGTGACCGTGACCCAGGACTCCTCCCTGCAGGACGGCGAGTTCATCTACAAGGTGAAGCTGCGCGGCACCAACTTCCCCTCCGACGGCCCCGTAATGCAGAAGAAGACCATGGGCTGGGAGGCCTCCTCCGAGCGGATGTACCCCGAGGACGGCGCCCTGAAGGGCGAGATCAAGCAGAGGCTGAAGCTGAAGGACGGCGGCCACTACGACGCTGAGGTCAAGACCACCTACAAGGCCAAGAAGCCCGTGCAGCTGCCCGGCGCCTACAACGTCAACATCAAGTTGGACATCACCTCCCACAACGAGGACTACACCATCGTGGAACAGTACGAACGCGCCGAGGGCCGCCACTCCACCGGCGGCATGGACGAGCTGTACAAGGGCAGCGCCGGCAGCGCCGCCGGCAGCGGCGAGTTCATGTCTAAAGGGGAAGAATTATTTACTGGGGTAGTACCTATATTAGTAGAATTAGACGGGGACGTAAATGGGCATAAATTTTCTGTATCTGGGGAAGGGGAAGGGGACGCAACTTATGGGAAATTAACTTTAAAATTTATATGTACTACTGGGAAATTACCTGTACCTTGGCCTACTTTAGTAACTACTTTAACTTATGGGGTACAATGTTTTTCTAGATATCCTGACCATATGAAACAACATGACTTTTTTAAATCTGCAATGCCTGAAGGGTATGTACAAGAAAGAACTATATTTTTTAAAGACGACGGGAATTATAAAACTAGAGCAGAAGTAAAATTTGAAGGGGACACTTTAGTAAATAGAATAGAATTAAAAGGGATAGACTTTAAAGAAGACGGGAATATATTAGGGCATAAATTAGAATATAATTATAATTCTCATAATGTATATATAATGGCAGACAAACAAAAAAATGGGATAAAAGTAAATTTTAAAATAAGACATAATATAGAAGACGGGTCTGTACAATTAGCAGACCATTATCAACAAAATACTCCTATAGGGGACGGGCCTGTATTATTACCTGACAATCATTATTTATCTACTCAATCTGCATTATCTAAAGACCCTAATGAAAAAAGAGACCATATGGTATTATTAGAATTTGTAACTGCAGCAGGGATAACTTTAGGGATGGACGAATTATATAAATAA |
| **mGFP100Dv3** | ATGGTGAGCAAGGGCGAGGAGGATAACATGGCCATCATCAAGGAGTTCATGCGCTTCAAGGTGCACATGGAGGGCTCCGTGAACGGCCACGAGTTCGAGATCGAGGGCGAGGGCGAGGGACGTCCGTATGAAGGAACGCAAACGGCTAAATTGAAAGTCACGAAAGGAGGACCGTTGCCGTTTGCTTGGGATATTTTGAGCCCACAATTTATGTATGGAAGCAAAGCTTATGTCAAGCACCCCGCCGACATCCCCGACTACTTGAAGCTGTCCTTCCCCGAGGGCTTCAAGTGGGAGCGCGTGATGAACTTCGAGGACGGCGGCGTGGTGACCGTGACCCAGGACTCCTCCCTGCAGGACGGCGAGTTCATCTACAAGGTGAAGCTGCGCGGCACCAACTTCCCCTCCGACGGCCCCGTAATGCAGAAGAAGACCATGGGCTGGGAGGCCTCCTCCGAGCGGATGTACCCCGAGGACGGCGCCCTGAAGGGCGAGATCAAGCAGAGGCTGAAGCTGAAGGACGGCGGCCACTACGACGCTGAGGTCAAGACCACCTACAAGGCCAAGAAGCCCGTGCAGCTGCCCGGCGCCTACAACGTCAACATCAAGTTGGACATCACCTCCCACAACGAGGACTACACCATCGTGGAACAGTACGAACGCGCCGAGGGCCGCCACTCCACCGGCGGCATGGACGAGCTGTACAAGGGCAGCGCCGGCAGCGCCGCCGGCAGCGGCGAGTTCATGTCTAAAGGGGAAGAATTATTTACTGGGGTAGTACCTATATTAGTAGAATTAGACGGGGACGTAAATGGGCATAAATTTTCTGTATCTGGGGAAGGGGAAGGGGACGCAACTTATGGGAAATTAACTTTAAAATTTATATGTACTACTGGGAAATTACCTGTACCTTGGCCTACTTTAGTAACTACTTTAACTTATGGGGTACAATGTTTTTCTAGATATCCTGACCATATGAAACAACATGACTTTTTTAAATCTGCAATGCCTGAAGGGTATGTACAAGAAAGAACTATATTTTTTAAAGACGACGGGAATTATAAAACTAGAGCAGAAGTAAAATTTGAAGGGGACACTTTAGTAAATAGAATAGAATTAAAAGGGATAGACTTTAAAGAAGACGGGAATATATTAGGGCATAAATTAGAATATAATTATAATTCTCATAATGTATATATAATGGCAGACAAACAAAAAAATGGGATAAAAGTAAATTTTAAAATAAGACATAATATAGAAGACGGGTCTGTACAATTAGCAGACCATTATCAACAAAATACTCCTATAGGGGACGGGCCTGTATTATTACCTGACAATCATTATTTATCTACTCAATCTGCATTATCTAAAGACCCTAATGAAAAAAGAGACCATATGGTATTATTAGAATTTGTAACTGCAGCAGGGATAACTTTAGGGATGGACGAATTATATAAATAA |
| **mGFP100Dv4** | ATGGTGAGCAAGGGCGAGGAGGATAACATGGCCATCATCAAGGAGTTCATGCGCTTCAAGGTGCACATGGAGGGCTCCGTGAACGGCCACGAGTTCGAGATCGAGGGCGAGGGCGAGGGCCGCCCCTACGAGGGCACCCAGACCGCCAAGCTGAAGGTGACCAAGGGTGGCCCCCTGCCCTTCGCCTGGGACATCCTGTCCCCTCAGTTCATGTACGGCTCCAAGGCCTACGTGAAACATCCGGCTGATATTCCGGATTATCTGAAATTGAGCTTTCCGGAAGGATTTAAATGGGAACGTGTCATGAATTTTGAAGATGGAGGAGTCGTCACGGTCACGCAAGATAGCAGCTTGCAGGACGGCGAGTTCATCTACAAGGTGAAGCTGCGCGGCACCAACTTCCCCTCCGACGGCCCCGTAATGCAGAAGAAGACCATGGGCTGGGAGGCCTCCTCCGAGCGGATGTACCCCGAGGACGGCGCCCTGAAGGGCGAGATCAAGCAGAGGCTGAAGCTGAAGGACGGCGGCCACTACGACGCTGAGGTCAAGACCACCTACAAGGCCAAGAAGCCCGTGCAGCTGCCCGGCGCCTACAACGTCAACATCAAGTTGGACATCACCTCCCACAACGAGGACTACACCATCGTGGAACAGTACGAACGCGCCGAGGGCCGCCACTCCACCGGCGGCATGGACGAGCTGTACAAGGGCAGCGCCGGCAGCGCCGCCGGCAGCGGCGAGTTCATGTCTAAAGGGGAAGAATTATTTACTGGGGTAGTACCTATATTAGTAGAATTAGACGGGGACGTAAATGGGCATAAATTTTCTGTATCTGGGGAAGGGGAAGGGGACGCAACTTATGGGAAATTAACTTTAAAATTTATATGTACTACTGGGAAATTACCTGTACCTTGGCCTACTTTAGTAACTACTTTAACTTATGGGGTACAATGTTTTTCTAGATATCCTGACCATATGAAACAACATGACTTTTTTAAATCTGCAATGCCTGAAGGGTATGTACAAGAAAGAACTATATTTTTTAAAGACGACGGGAATTATAAAACTAGAGCAGAAGTAAAATTTGAAGGGGACACTTTAGTAAATAGAATAGAATTAAAAGGGATAGACTTTAAAGAAGACGGGAATATATTAGGGCATAAATTAGAATATAATTATAATTCTCATAATGTATATATAATGGCAGACAAACAAAAAAATGGGATAAAAGTAAATTTTAAAATAAGACATAATATAGAAGACGGGTCTGTACAATTAGCAGACCATTATCAACAAAATACTCCTATAGGGGACGGGCCTGTATTATTACCTGACAATCATTATTTATCTACTCAATCTGCATTATCTAAAGACCCTAATGAAAAAAGAGACCATATGGTATTATTAGAATTTGTAACTGCAGCAGGGATAACTTTAGGGATGGACGAATTATATAAATAA |
| **mGFP100Dv5** | ATGGTGAGCAAGGGCGAGGAGGATAACATGGCCATCATCAAGGAGTTCATGCGCTTCAAGGTGCACATGGAGGGCTCCGTGAACGGCCACGAGTTCGAGATCGAGGGCGAGGGCGAGGGCCGCCCCTACGAGGGCACCCAGACCGCCAAGCTGAAGGTGACCAAGGGTGGCCCCCTGCCCTTCGCCTGGGACATCCTGTCCCCTCAGTTCATGTACGGCTCCAAGGCCTACGTGAAGCACCCCGCCGACATCCCCGACTACTTGAAGCTGTCCTTCCCCGAGGGCTTCAAGTGGGAGCGCGTGATGAACTTCGAGGACGGCGGCGTGGTGACCGTGACCCAGGACTCCTCCCTGCAAGATGGAGAATTTATTTATAAAGTCAAATTGCGTGGAACGAATTTTCCGAGCGATGGACCGGTGATGCAAAAAAAAACGATGGGATGGGAAGCTAGCAGCGAACGCATGTATCCGGAGGACGGCGCCCTGAAGGGCGAGATCAAGCAGAGGCTGAAGCTGAAGGACGGCGGCCACTACGACGCTGAGGTCAAGACCACCTACAAGGCCAAGAAGCCCGTGCAGCTGCCCGGCGCCTACAACGTCAACATCAAGTTGGACATCACCTCCCACAACGAGGACTACACCATCGTGGAACAGTACGAACGCGCCGAGGGCCGCCACTCCACCGGCGGCATGGACGAGCTGTACAAGGGCAGCGCCGGCAGCGCCGCCGGCAGCGGCGAGTTCATGTCTAAAGGGGAAGAATTATTTACTGGGGTAGTACCTATATTAGTAGAATTAGACGGGGACGTAAATGGGCATAAATTTTCTGTATCTGGGGAAGGGGAAGGGGACGCAACTTATGGGAAATTAACTTTAAAATTTATATGTACTACTGGGAAATTACCTGTACCTTGGCCTACTTTAGTAACTACTTTAACTTATGGGGTACAATGTTTTTCTAGATATCCTGACCATATGAAACAACATGACTTTTTTAAATCTGCAATGCCTGAAGGGTATGTACAAGAAAGAACTATATTTTTTAAAGACGACGGGAATTATAAAACTAGAGCAGAAGTAAAATTTGAAGGGGACACTTTAGTAAATAGAATAGAATTAAAAGGGATAGACTTTAAAGAAGACGGGAATATATTAGGGCATAAATTAGAATATAATTATAATTCTCATAATGTATATATAATGGCAGACAAACAAAAAAATGGGATAAAAGTAAATTTTAAAATAAGACATAATATAGAAGACGGGTCTGTACAATTAGCAGACCATTATCAACAAAATACTCCTATAGGGGACGGGCCTGTATTATTACCTGACAATCATTATTTATCTACTCAATCTGCATTATCTAAAGACCCTAATGAAAAAAGAGACCATATGGTATTATTAGAATTTGTAACTGCAGCAGGGATAACTTTAGGGATGGACGAATTATATAAATAA |
| **mGFP100Dv6** | ATGGTGAGCAAGGGCGAGGAGGATAACATGGCCATCATCAAGGAGTTCATGCGCTTCAAGGTGCACATGGAGGGCTCCGTGAACGGCCACGAGTTCGAGATCGAGGGCGAGGGCGAGGGCCGCCCCTACGAGGGCACCCAGACCGCCAAGCTGAAGGTGACCAAGGGTGGCCCCCTGCCCTTCGCCTGGGACATCCTGTCCCCTCAGTTCATGTACGGCTCCAAGGCCTACGTGAAGCACCCCGCCGACATCCCCGACTACTTGAAGCTGTCCTTCCCCGAGGGCTTCAAGTGGGAGCGCGTGATGAACTTCGAGGACGGCGGCGTGGTGACCGTGACCCAGGACTCCTCCCTGCAGGACGGCGAGTTCATCTACAAGGTGAAGCTGCGCGGCACCAACTTCCCCTCCGACGGCCCCGTAATGCAGAAGAAGACCATGGGCTGGGAGGCCTCCTCCGAGCGGATGTACCCCGAAGATGGAGCTTTGAAAGGAGAAATTAAACAACGCTTGAAATTGAAAGATGGAGGACATTATGATGCCGAAGTGAAAACGACGTATAAAGCTAAAAAACCGGTCCAATTGCCGGGAGCCTACAACGTCAACATCAAGTTGGACATCACCTCCCACAACGAGGACTACACCATCGTGGAACAGTACGAACGCGCCGAGGGCCGCCACTCCACCGGCGGCATGGACGAGCTGTACAAGGGCAGCGCCGGCAGCGCCGCCGGCAGCGGCGAGTTCATGTCTAAAGGGGAAGAATTATTTACTGGGGTAGTACCTATATTAGTAGAATTAGACGGGGACGTAAATGGGCATAAATTTTCTGTATCTGGGGAAGGGGAAGGGGACGCAACTTATGGGAAATTAACTTTAAAATTTATATGTACTACTGGGAAATTACCTGTACCTTGGCCTACTTTAGTAACTACTTTAACTTATGGGGTACAATGTTTTTCTAGATATCCTGACCATATGAAACAACATGACTTTTTTAAATCTGCAATGCCTGAAGGGTATGTACAAGAAAGAACTATATTTTTTAAAGACGACGGGAATTATAAAACTAGAGCAGAAGTAAAATTTGAAGGGGACACTTTAGTAAATAGAATAGAATTAAAAGGGATAGACTTTAAAGAAGACGGGAATATATTAGGGCATAAATTAGAATATAATTATAATTCTCATAATGTATATATAATGGCAGACAAACAAAAAAATGGGATAAAAGTAAATTTTAAAATAAGACATAATATAGAAGACGGGTCTGTACAATTAGCAGACCATTATCAACAAAATACTCCTATAGGGGACGGGCCTGTATTATTACCTGACAATCATTATTTATCTACTCAATCTGCATTATCTAAAGACCCTAATGAAAAAAGAGACCATATGGTATTATTAGAATTTGTAACTGCAGCAGGGATAACTTTAGGGATGGACGAATTATATAAATAA |
| **mGFP100Dv7** | ATGGTGAGCAAGGGCGAGGAGGATAACATGGCCATCATCAAGGAGTTCATGCGCTTCAAGGTGCACATGGAGGGCTCCGTGAACGGCCACGAGTTCGAGATCGAGGGCGAGGGCGAGGGCCGCCCCTACGAGGGCACCCAGACCGCCAAGCTGAAGGTGACCAAGGGTGGCCCCCTGCCCTTCGCCTGGGACATCCTGTCCCCTCAGTTCATGTACGGCTCCAAGGCCTACGTGAAGCACCCCGCCGACATCCCCGACTACTTGAAGCTGTCCTTCCCCGAGGGCTTCAAGTGGGAGCGCGTGATGAACTTCGAGGACGGCGGCGTGGTGACCGTGACCCAGGACTCCTCCCTGCAGGACGGCGAGTTCATCTACAAGGTGAAGCTGCGCGGCACCAACTTCCCCTCCGACGGCCCCGTAATGCAGAAGAAGACCATGGGCTGGGAGGCCTCCTCCGAGCGGATGTACCCCGAGGACGGCGCCCTGAAGGGCGAGATCAAGCAGAGGCTGAAGCTGAAGGACGGCGGCCACTACGACGCTGAGGTCAAGACCACCTACAAGGCCAAGAAGCCCGTGCAGCTGCCCGGCGCTTATAATGTGAATATTAAACTGGATATTACGAGCCATAATGAAGATTATACGATTGTCGAGCAATATGAGCGTGCTGAAGGACGTCATAGCACGGGAGGAATGGATGAATTGTATAAAGGCAGCGCCGGCAGCGCCGCCGGCAGCGGCGAGTTCATGTCTAAAGGGGAAGAATTATTTACTGGGGTAGTACCTATATTAGTAGAATTAGACGGGGACGTAAATGGGCATAAATTTTCTGTATCTGGGGAAGGGGAAGGGGACGCAACTTATGGGAAATTAACTTTAAAATTTATATGTACTACTGGGAAATTACCTGTACCTTGGCCTACTTTAGTAACTACTTTAACTTATGGGGTACAATGTTTTTCTAGATATCCTGACCATATGAAACAACATGACTTTTTTAAATCTGCAATGCCTGAAGGGTATGTACAAGAAAGAACTATATTTTTTAAAGACGACGGGAATTATAAAACTAGAGCAGAAGTAAAATTTGAAGGGGACACTTTAGTAAATAGAATAGAATTAAAAGGGATAGACTTTAAAGAAGACGGGAATATATTAGGGCATAAATTAGAATATAATTATAATTCTCATAATGTATATATAATGGCAGACAAACAAAAAAATGGGATAAAAGTAAATTTTAAAATAAGACATAATATAGAAGACGGGTCTGTACAATTAGCAGACCATTATCAACAAAATACTCCTATAGGGGACGGGCCTGTATTATTACCTGACAATCATTATTTATCTACTCAATCTGCATTATCTAAAGACCCTAATGAAAAAAGAGACCATATGGTATTATTAGAATTTGTAACTGCAGCAGGGATAACTTTAGGGATGGACGAATTATATAAATAA |
| **mGFP100Dv8** | ATGGTGAGCAAGGGCGAGGAGGATAACATGGCCATCATCAAGGAGTTCATGCGCTTCAAGGTGCACATGGAGGGCTCCGTGAACGGCCACGAGTTCGAGATCGAGGGCGAGGGCGAGGGCCGCCCCTACGAGGGCACCCAGACCGCCAAGCTGAAGGTGACCAAGGGTGGCCCCCTGCCCTTCGCCTGGGACATCCTGTCCCCTCAGTTCATGTACGGCTCCAAGGCCTACGTGAAGCACCCCGCCGACATCCCCGACTACTTGAAGCTGTCCTTCCCCGAGGGCTTCAAGTGGGAGCGCGTGATGAACTTCGAGGACGGCGGCGTGGTGACCGTGACCCAGGACTCCTCCCTGCAGGACGGCGAGTTCATCTACAAGGTGAAGCTGCGCGGCACCAACTTCCCCTCCGACGGCCCCGTAATGCAGAAGAAGACCATGGGCTGGGAGGCCTCCTCCGAGCGGATGTACCCCGAGGACGGCGCCCTGAAGGGCGAGATCAAGCAGAGGCTGAAGCTGAAGGACGGCGGCCACTACGACGCTGAGGTCAAGACCACCTACAAGGCCAAGAAGCCCGTGCAGCTGCCCGGCGCCTACAACGTCAACATCAAGTTGGACATCACCTCCCACAACGAGGACTACACCATCGTGGAACAGTACGAACGCGCCGAGGGCCGCCACTCCACCGGCGGCATGGACGAGCTGTACAAGGGCAGCGGCGAGGGCCGCGGCTCCCTGCTGACCTGCGGCGATGTGGAGGAGAACCCCGGCCCCATGTCTAAAGGGGAAGAATTATTTACTGGGGTAGTACCTATATTAGTAGAATTAGACGGGGACGTAAATGGGCATAAATTTTCTGTATCTGGGGAAGGGGAAGGGGACGCAACTTATGGGAAATTAACTTTAAAATTTATATGTACTACTGGGAAATTACCTGTACCTTGGCCTACTTTAGTAACTACTTTAACTTATGGGGTACAATGTTTTTCTAGATATCCTGACCATATGAAACAACATGACTTTTTTAAATCTGCAATGCCTGAAGGGTATGTACAAGAAAGAACTATATTTTTTAAAGACGACGGGAATTATAAAACTAGAGCAGAAGTAAAATTTGAAGGGGACACTTTAGTAAATAGAATAGAATTAAAAGGGATAGACTTTAAAGAAGACGGGAATATATTAGGGCATAAATTAGAATATAATTATAATTCTCATAATGTATATATAATGGCAGACAAACAAAAAAATGGGATAAAAGTAAATTTTAAAATAAGACATAATATAGAAGACGGGTCTGTACAATTAGCAGACCATTATCAACAAAATACTCCTATAGGGGACGGGCCTGTATTATTACCTGACAATCATTATTTATCTACTCAATCTGCATTATCTAAAGACCCTAATGAAAAAAGAGACCATATGGTATTATTAGAATTTGTAACTGCAGCAGGGATAACTTTAGGGATGGACGAATTATATAAATAA |
| **RpL10Aa Endo** | ATGGACTACAAAGACCATGACGGTGATTATAAAGATCATGACATCGATTACAAGGATGACGATGACAAGGTGTCGAAAGTTTCTCGCGATACGATTTACGTTGCAGTCAAAAATATCCTGCTGAACTCGCAGGCCAAAGGACCAGACTGCCTGGAGACGGTGGAGCTGCAGATTGGGCTGAGGGATTATGATCCTGACAAATGCAAGCGGTTCCATGGAAGTGTACTATTGCATCACCTGGCGGTTCCACAACTAAAGGTCTGCGTCTTCGGGGATCAGGAGCACTGTTATAAGGCCAAAGCCATAGGAGTTGATTGCCTAGATGTGGAGGCTTTGAAAAAGCTGAACAAAGATCCCAAGTTGACAAAGAAGTTGTCCAAAGCTTACGATGTCTTCCTGGCCTCCGAATCGATAATTAAGCAGATCCCAAGGCTACTGGGTCCTGGTCTCACCAATGCGGGCAAATTTCTTACTCCTTTGGCTCGTGGCGAATCTATGAGTTCCAAAATCAAAATACTATCTACCAAAAAGAAGCATATGAAAAGGATGGAATGTCTTTCCGTTAATGTTGGCCATGTTGGCATGCACCCAGAGGAACTAGCTCGAAACATAGCAATATCGATCAACTTTTTAGTGTCCTTGCTGAAGGATAACTGGCAGAATGTGCGCTCACTTCATATAAAATCATCGTTGGGCGTACCTCATCAGCTCTATTGA |
| **RpL10Aa Com** | ATGGACTACAAAGACCATGACGGTGATTATAAAGATCATGACATCGATTACAAGGATGACGATGACAAGGTGAGCAAGGTGAGCCGCGATACCATCTACGTGGCCGTGAAGAACATCCTGCTGAACAGCCAGGCCAAGGGCCCCGATTGCCTGGAGACCGTGGAGCTGCAGATCGGCCTGCGCGATTACGATCCCGATAAGTGCAAGCGCTTCCACGGCAGCGTGCTGCTGCACCACCTGGCCGTGCCCCAGCTGAAGGTGTGCGTGTTCGGCGATCAGGAGCACTGCTACAAGGCCAAGGCCATCGGCGTGGATTGCCTGGATGTGGAGGCCCTGAAGAAGCTGAACAAGGATCCCAAGCTGACCAAGAAGCTGAGCAAGGCCTACGATGTGTTCCTGGCCAGCGAGAGCATCATCAAGCAGATCCCCCGCCTGCTGGGCCCCGGCCTGACCAACGCCGGCAAGTTCCTGACCCCCCTGGCCCGCGGCGAGAGCATGAGCAGCAAGATCAAGATCCTGAGCACCAAGAAGAAGCACATGAAGCGCATGGAGTGCCTGAGCGTGAACGTGGGCCACGTGGGCATGCACCCCGAGGAGCTGGCCCGCAACATCGCCATCAGCATCAACTTCCTGGTGAGCCTGCTGAAGGATAACTGGCAGAACGTGCGCAGCCTGCACATCAAGAGCAGCCTGGGCGTGCCCCACCAGCTGTACTAA |
