# Supplementary material for: Distinct responses to rare codons in select Drosophila tissues
Source: eLife. 2022 May 6;11:e76893. doi: 10.7554/eLife.76893 (PMC9116940; doi:10.7554/eLife.76893)
Supplement: Supplementary file 2. [file elife-76893-supp2.docx]

**Supplementary File 2 – Occurrences of codons known to impair translation do not explain reporter expression patterns.**

| **Reporter Name** | **Count CGA codons** | **Count AGG codons** | **Count AGG-AGG codon pairs** |
| --- | --- | --- | --- |
| **GFP0D** | 0 | 0 | 0 |
| **GFP30D** | 1 | 1 | 0 |
| **GFP50D** | 1 | 1 | 0 |
| **GFP60Dv1** | 0 | 1 | 0 |
| **GFP60Dv2** | 1 | 0 | 0 |
| **GFP60Dv3** | 1 | 0 | 0 |
| **GFP70D** | 1 | 0 | 0 |
| **GFP80D** | 0 | 0 | 0 |
| **GFP90D** | 0 | 0 | 0 |
| **GFP100D** | 0 | 0 | 0 |
| **GFP50C3'** | 0 | 0 | 0 |
| **GFP60C3'** | 0 | 0 | 0 |
| **GFP70C3'** | 0 | 0 | 0 |
| **GFP80C3'** | 0 | 0 | 0 |
| **GFP90C3'** | 0 | 0 | 0 |
| **GFP50C5'** | 0 | 1 | 0 |
| **GFP54C3'** | 0 | 0 | 0 |
| **mGFP100Dv1** | 0 | 1 | 0 |
| **mGFP100Dv2** | 0 | 1 | 0 |
| **mGFP100Dv3** | 0 | 1 | 0 |
| **mGFP100Dv4** | 0 | 1 | 0 |
| **mGFP100Dv5** | 0 | 1 | 0 |
| **mGFP100Dv6** | 0 | 0 | 0 |
| **mGFP100Dv7** | 0 | 1 | 0 |
| **RpL10Aa Endo** | 1 | 3 | 0 |
| **RpL10Aa Com** | 0 | 0 | 0 |
