# Supplementary material for: Distinct responses to rare codons in select Drosophila tissues
Source: eLife. 2022 May 6;11:e76893. doi: 10.7554/eLife.76893 (PMC9116940; doi:10.7554/eLife.76893)
Supplement: Supplementary file 3. [file elife-76893-supp3.docx]

| ***Ubi-p63E* promoter plus 5’ UTRs contained in pBID-Ubi plasmid**  **Supplementary File 3 – Sequences of pBID-Ubi plasmid promoter and UTRs.** | GGGGACCTAGATCCTTCACATAGATCTTGTCGCCGGAACGCAGCGACAGAGATTCCAATGTGTCCGTATCTTTCAGGCTTTTGCCCTTCAGTTCCAGACGAAGCGACTGGCGATTCGCGTGTGGGGTCTGCTTCAGGGTCTTGTGAATTAGGGCGCGCAGATCGCCGATGGGCGTGGCGCCGGAGGGCACCTTCACCTTGCCGTACGGCTTGCTGTTCTTCGCGTTCAAAATCTCCAGCTCCATTTTGCTTTCGGTGCGCTTGCAATCAGTACTGTCCAAAATCGAAAATCGCCGAACCGTAGTGTGACCGTGCGGGGCTCTGCGAAAATAAACTTTTTTAGGTATATGGCCACACACGGGGAAAGCACAGTGGATTATATGTTTTAATATTATAATATGCAGGTTTTCATTACTTATCCAGATGTAAGCCCACTTAAAGCGATTTAACAATTATTTGCCGAAAGAGTATAAACAAATTTCACTTAAAAATGGATTAAGAAAAGCTTGTGTAAGATTATGCGCAGCGTTGCCAGATAGCTCCATTTAAAACACTTCAAAAACAATAAGTTTTGAAAATATATACATAAATAGCAGTCGTTGCCGCAACGCTCAACACATCACACTTTTAAAACACCCTTTACCTACACAGAATTACTTTTTAAATTTCCAGTCAAGCTGCGAGTTTCAAAATTATAGCCGGTAGAGAAGACAGTGCTATTTCAAAAGCAAACTAACAAGGGTCTTAAATTCCAAAACACCAATCCTAACAAGCCTTGGACTTTTGTAAGTTTAGATCAAAGGTGGCATTGCATTCAATGTCATGGTAAGAAGTAGGTCGTCTAGGTAGAAATCCTCATTCAGCCGGTCAAGTCAGTACGAGAAAGGTCTCAATTTGAAATTGTCTTAAAAATATTTTATTGTTTTGTACTGTGGTGAGTTTAAACGAAAAACACAAAAAAAAAGTGATACACAGAAATCATAAAAAATTTTAATACAAGGTATTCGTACGTATCAAAAACATTTCGGCACAATTTTTTTTCTCTGTACTAAAGTGTTACGAACACTACGGTATTTTTTAGTGATTTTCAACGGACACCGAAGGTATATAAACAGCGTTCGCGAACGGTCGCCTTCAAAACCAATTGACATTTGCAGCAGCAAGTACAAGTAGAAAGTAAAGCGCAATCAGCGAAAAATTTATACTTAATTGTTGGTGATTAAAGTACAATTAAAAGAACATTCTCGAAAGTCACAAGAAACGTAAGTTTTTAACTCGCTGTTACCAATTAGTAATAAGAGCAACAAGACGTTGAGTAATTTCAAGAAAAACTGCATTTCAAGGTCTTTGTTCGGCCATTTTTTTTTTATTCAACGCTCTACGTAATTACAAAATAAGAAATTGGCAGCCACGCATCTTGTTTTCCCAATGAATTGGCATCAAAACGCAAACAAATCTATAAATAAAACTTGCGTGTTGATTTTCGCCAAGATTTATTGGCAAATTGTGAAATTCGCAGTGACGCATTTGAAAATTCGAGAAATCACGAACGCACTCGAGCATTTGTGTGCATGTTATTAGTTAGTTGTTTAGTTAATTGAAGTATTTTACCAACGAAATCCACTTATTTTTAGCTGAAATAGAGTAGGTTGCTTAAACAAAGCCACGTCTGAAAATTTCTTATTGCTTGTAGTTGTGACGTCACCATATACACACAAAATAATGTGTATGCATGCATTTCAGCTGTGTATATATACATGCACACACTCGCAACACGAAAACGATGACGAAGCAACGGAACAAAGGTTTCTCAACTACCCTTTGTTCCCTGTTTCTTCGCTTTCCTTTGTTCCAATATTCGTAGAGGGTTAATAGGGGTTTCTCAACAAAGTTGGCGTCGATAAATAAGTTTCCCATTTTTATTCCCCAGCCAGGAAGTTAGTTTCAATAGTTTTGTAATTTCAACGAAACTCATTTGATTTCGTACTAATTTTCCACATCTCTATTTTCTGCCCGCA |
| --- | --- |
| **Transgene 5’ UTR RD isoform recovered from testis and ovary** | TTTGCAGCAGCAAGTACAAGTAGAAAGTAAAGCGCAATCAGCGAAAAATTTATACTTAATTGTTGGTGATTAAAGTACAATTAAAAGAACATTCTCGAAAGTCACAAGAAACGCGGCCGCGGCTCGAGAAGGTACCGGTCACC |
| **Transgene 5’ UTR RC isoform recovered from testis and ovary** | TCAGCTGTGTATATATACATGCACACACTCGCAACACGAAAACGATGACGAAGCAACGGAACAAAGGTTTCTCAACTACCCTTTGTTCCCTGTTTCTTCGCTTTCCTTTGTTCCAATATTCGTAGAGGGTTAATAGGGGTTTCTCAACAAAGTTGGCGTCGATAAATAAGTTTCCCATTTTTATTCCCCAGCCAGGAAGTTAGTTTCAATAGTTTTGTAATTTCAACGAAACTCATTTGATTTCGTACTAATTTTCCACATCTCTATTTTCTGCCCGCAGCGGCCGCGGCTCGAGAAGGTACCGGTCACC |
| **Transgene 3’ UTR recovered from whole animal** | GGTACCTCTTAATTAACTGGCCTCATGGGCACCGGTCTAGAAGATCTTGGCCACGTAATAAGTGTGCGTTGAATTTATTCGCAAAAACATTGCATATTTTCGGCAAAGTAAAATTTTGTTGCATACCTTATCAAAAAATAAGTGCTGCATACTTTTTAGAGAAACCAAATAATTTTTTATTGCATACCCGTTTTTAATAAAATACATTGCATACCCTCTTTTAATAAAAAATATTGCATACTTTGACGAAACAAATTTTCGTTGCATACCCAATAAAAGATTATTATATTGCATACCCGTTTTTAATAAAATACATTGCATACCCTCTTTTAATAAAAAA |
