# Supplementary material for: Distinct responses to rare codons in select Drosophila tissues
Source: eLife. 2022 May 6;11:e76893. doi: 10.7554/eLife.76893 (PMC9116940; doi:10.7554/eLife.76893)
Supplement: Supplementary file 4. [file elife-76893-supp4.docx]

**Supplementary File 4 – Identity of child genes that gained max expression in the testis.**

| **Child Gene** | **Highest Expressed in Testis?** | **Parent Gene** | **Highest Expressed in Testis?** |
| --- | --- | --- | --- |
| FBgn0030215 | TRUE | FBgn0036726 | FALSE |
| FBgn0038281 | TRUE | FBgn0036213 | FALSE |
| FBgn0035585 | TRUE | FBgn0016119 | FALSE |
| FBgn0051952 | TRUE | FBgn0265523 | FALSE |
| FBgn0034822 | TRUE | FBgn0030616 | FALSE |
| FBgn0031345 | TRUE | FBgn0030329 | FALSE |
| FBgn0261395 | TRUE | FBgn0261394 | FALSE |
| FBgn0034205 | TRUE | FBgn0031456 | FALSE |
| FBgn0036212 | TRUE | FBgn0023177 | FALSE |
| FBgn0029728 | TRUE | FBgn0030459 | FALSE |
| FBgn0028513 | TRUE | FBgn0010497 | FALSE |
| FBgn0052971 | TRUE | FBgn0040339 | FALSE |
| FBgn0051924 | TRUE | FBgn0019982 | FALSE |
| FBgn0036497 | TRUE | FBgn0020255 | FALSE |
| FBgn0265606 | TRUE | FBgn0004066 | FALSE |
| FBgn0015008 | TRUE | FBgn0025633 | FALSE |
| FBgn0036747 | TRUE | FBgn0031170 | FALSE |
| FBgn0034651 | TRUE | FBgn0264694 | FALSE |
| FBgn0039576 | TRUE | FBgn0030733 | FALSE |
| FBgn0050075 | TRUE | FBgn0003401 | FALSE |
| FBgn0032439 | TRUE | FBgn0010774 | FALSE |
| FBgn0053017 | TRUE | FBgn0035253 | FALSE |
| FBgn0034837 | TRUE | FBgn0015288 | FALSE |
| FBgn0250904 | TRUE | FBgn0033603 | FALSE |
| FBgn0030377 | TRUE | FBgn0015622 | FALSE |
| FBgn0039622 | TRUE | FBgn0015218 | FALSE |
| FBgn0038486 | TRUE | FBgn0037440 | FALSE |
| FBgn0030979 | TRUE | FBgn0036717 | FALSE |
| FBgn0053300 | TRUE | FBgn0052602 | FALSE |
| FBgn0036496 | TRUE | FBgn0025790 | FALSE |
| FBgn0052847 | TRUE | FBgn0030693 | FALSE |
| FBgn0051477 | TRUE | FBgn0014391 | FALSE |
| FBgn0264077 | TRUE | FBgn0015622 | FALSE |
| FBgn0034937 | TRUE | FBgn0262699 | FALSE |
| FBgn0039371 | TRUE | FBgn0033906 | FALSE |
| FBgn0038209 | TRUE | FBgn0013305 | FALSE |
| FBgn0015025 | TRUE | FBgn0039860 | FALSE |
| FBgn0015520 | TRUE | FBgn0004227 | FALSE |
| FBgn0032680 | TRUE | FBgn0031145 | FALSE |
| FBgn0031504 | TRUE | FBgn0039970 | FALSE |
| FBgn0031856 | TRUE | FBgn0024991 | FALSE |
| FBgn0052110 | TRUE | FBgn0027603 | FALSE |
| FBgn0030937 | TRUE | FBgn0034098 | FALSE |
| FBgn0050354 | TRUE | FBgn0260008 | FALSE |
