# Supplementary material for: Distinct responses to rare codons in select Drosophila tissues
Source: eLife. 2022 May 6;11:e76893. doi: 10.7554/eLife.76893 (PMC9116940; doi:10.7554/eLife.76893)
Supplement: Supplementary file 5. [file elife-76893-supp5.docx]

**Supplementary File 5 – Primer sequences for qRT-PCR and RT-PCR experiments.**

| **Target** | **Direction** | **Primer Sequence 5'-3'** |
| --- | --- | --- |
| 0D | F | GGCAAGCTGACCCTGAAGTT |
| 0D | R | TTCATGTGATCGGGGTAGCG |
| 30D | F | ATTCTCAGTGTCGGGCGAAG |
| 30D | R | AGTAACCAGTGTTGGCCAGG |
| 50D | F | TTTTCAGTGTCGGGCGAAGG |
| 50D | R | TAAAGTCGGCCAAGGGACAG |
| 60Dv1 | F | TCGACGGTGATGTAAATGGGC |
| 60Dv1 | R | ATTTCCCGTATGTGGCGTCC |
| 60Dv2 | F | CGGGGATGTCAATGGTCAC |
| 60Dv2 | R | GTAGGCCAAGGTACAGGC |
| 60Dv3 | F | TACTCCAATAGGGGACGGGC |
| 60Dv3 | R | TCCCGAGAGTTATCCCTGC |
| 70D | F | ATTACCTGTACCTTGGCCCAC |
| 70D | R | TCCTGAACATAGCCTTCTGGC |
| 80D | F | TTAGACGGAGACGTGAATGGG |
| 80D | R | TTCCCATAAGTTGCGTCCCC |
| 90D | F | ATTACCTGTACCTTGGCCGAC |
| 90D | R | CGTGTTGTTTCATGTGGTCAGG |
| mGFP100Dv1, 100D, & 50C5' | F | AGGGGAAGAATTATTTACTGGGGT |
| mGFP100Dv1, 100D, & 50C5' | R | CCCATAAGTTGCGTCCCCTT |
| 50C3'-90C3' | F | TACTCCTATAGGGGACGGGC |
| 50C3'-90C3' | R | TCCATCCCTAAAGTTATCCCTGC |
| RpL10Aa Endo | F | TTACTCCTTTGGCTCGTGGC |
| RpL10Aa Endo | R | CGAGCTAGTTCCTCTGGGTG |
| RpL10Aa Com | F | GAAGCACATGAAGCGCATGG |
| RpL10Aa Com | R | CGCACGTTCTGCCAGTTATC |
| 5' UTR species RA | F | CACCCTTTACCTACACAGAATTAC |
| 5' UTR species RB | F | CAGTACGAGAAAGGTCTC |
| 5' UTR species RB/D | F | GCAGCAGCAAGTACAAGTAG |
| 5' UTR species RC | F | CTCAACAAAGTTGGCGTCG |
| 5' UTR all species | R | CCTCGATCTCGAACTCGTG |
